# Plant-Derived Carotenoid Lutein Demonstrates Multifunctional Antiviral Activity against Influenza A Virus *in vitro*

**DOI:** 10.4014/jmb.2507.07017

**Published:** 2025-11-18

**Authors:** Ju Won Kim, Han-Sol Ryu, Sanghyun Lee, Sejin Jeon, Sun-Woo Yoon, Yo Han Jang

**Affiliations:** 1 Vaccine Biotechnology Major, Gyeongkuk National University, Andong 36729, Republic of Korea; 2 Department of Plant Science and Technology, Chung-Ang University, Anseong 17546, Republic of Korea

**Keywords:** Lutein, influenza virus, antiviral agent, virucidal activity, hemagglutinin, neuraminidase

## Abstract

Influenza virus infections remain a major global health concern, causing annual outbreaks with high morbidity and mortality. The emergence of drug resistance and adverse effects from existing antivirals underscores the need for new therapeutic agents. This study presents the first evaluation of the antiviral activity and mechanisms of the dietary carotenoid lutein against influenza viruses. Lutein exhibited strong virucidal activity against influenza A (IAV) and B (IBV) viruses, as well as Japanese encephalitis virus (JEV), but showed only weak effects against the non-enveloped rotavirus, suggesting a preference for enveloped viruses. Dynamic light scattering analysis revealed that lutein disrupted viral particle integrity, causing aggregation and a reduction in particle numbers. Functional assays further demonstrated that lutein inhibited the activities of viral hemagglutinin (HA) and neuraminidase (NA). Lutein also suppressed viral replication when applied to cells both before and after infection, indicating its prophylactic and therapeutic potential. Collectively, these findings demonstrate that lutein exerts multifunctional antiviral effects through virucidal activity, inhibition of HA and NA activity, and suppression of viral replication. As the first report to elucidate lutein’s multifaceted antiviral mechanisms, this study supports its potential as a natural antiviral candidate. *In vivo* studies will be essential to further assess its pharmacokinetics, efficacy, and safety for therapeutic applications against influenza.

## Introduction

Influenza viruses, belonging to the family *Orthomyxoviridae*, are classified into types A, B, C, and D, with influenza A and B viruses (IAVs and IBVs) being the primary human pathogens responsible for seasonal epidemics [1]. Influenza infections cause respiratory symptoms such as fever, cough, and sore throat, which can progress to severe complications like pneumonia [2]. IAVs possess a segmented, negative-sense RNA genome with high mutation rates, leading to antigenic drift and shift, and the emergence of diverse subtypes due to variations in surface glycoproteins HA and NA [2, 3]. Current antiviral strategies target NA, RNA polymerase, or the M2 ion channel [4]. NA inhibitors like zanamivir, laninamivir, oseltamivir, and peramivir block viral release but face limitations due to drug resistance and side effects [5, 6]. RNA polymerase inhibitors such as favipiravir, pimodivir, and baloxavir marboxil impede viral replication but have safety concerns [7, 8]. M2 inhibitors including amantadine and rimantadine are ineffective against influenza B and also face resistance issues [4, 9]. These limitations highlight the need for novel antiviral therapies.

Phytochemicals are naturally occurring plant-derived compounds, including carotenoids, polyphenols, polysaccharides, and saponins, and are known for a wide range of biological activities, including anti-inflammatory, anticancer, antimicrobial, antioxidant, and immune-modulating effects [10-12]. In particular, their antiviral properties have been demonstrated against various viruses, including influenza virus, dengue virus, hepatitis C virus, and herpes simplex virus, highlighting their potential as novel antiviral agents [13-16]. Our previous studies have shown that plant-derived extracts and phytochemicals have antiviral activity against influenza viruses through multiple mechanisms [17-20]. Lutein, a carotenoid pigment classified as a phytochemical, is abundant in red, yellow, and orange fruits and vegetables, as well as leafy greens such as spinach, kale, and yellow carrots [21]. Lutein has been reported to exhibit various biological effects, including skin protection, anticancer, anti-inflammatory, and anti-obesity activities, as well as the mitigation of lung and liver damage [21-23]. Additionally, lutein contributes to the prevention of cardiovascular diseases and exerts neuroprotective effects that may help in the management of neurodegenerative diseases, such as Alzheimer’s disease [24, 25]. It was demonstrated that lutein possessed antiviral activity against vaccinia virus, dengue virus, and hepatitis C virus [26-28]. However, antiviral activity of lutein against influenza viruses remains limited. A study reported that lutein was predicted to bind to interleukin-6, thereby possibly contributing to antiviral activity against influenza virus [29]. However, antiviral activity and underlying mechanisms of lutein against influenza virus have not been described *in vitro* yet. In this study, we evaluated the antiviral activity of lutein against influenza viruses, with specific focus on direct effects to the viral surface glycoproteins and viral membrane integrity. As the first report to address antiviral activity of lutein against influenza viruses, our study demonstrates that lutein can be a promising candidate for developing antiviral therapeutics against the viral infections.

## Materials and Methods

### Reagents, Animal Cell Lines, and Viruses

Lutein was obtained from the Natural Product Institute of Science and Technology (www.nist.re.kr) (Anseong, Republic of Korea). L-ascorbic acid and oseltamivir phosphate (OP) were purchased from Sigma-Aldrich (USA), and gallic acid from Biosesang (Republic of Korea). Epigallocatechin gallate (EGCG) was supplied by Hunan Sunshine Bio-tech (China). Madin-Darby canine kidney (MDCK), baby hamster kidney (BHK-21), and MA104 cells were obtained from the American Type Culture Collection and maintained in Minimum Essential Medium (Gibco, USA) supplemented with 10% fetal bovine serum (Hyclone, USA) and Zellshield^®^ antibiotic (Minerva Biolabs GmbH, Germany) under a humidified atmosphere of 5% CO_2_ at 37°C. The following IAV strains were used: A/Puerto Rico/8/34 (A/PR8, H1N1), A/aquatic bird/Korea/w81/05 (A/MA81, H5N2), and an A/PR8-GFP reporter virus [30]. IBV strain B/Yamagata/16/88 (B/YAM16) was also used in this study. Influenza virus stocks were prepared by infecting MDCK cells at a multiplicity of infection (MOI) of 0.01, followed by harvesting and clarification of the supernatants by centrifugation. Japanese encephalitis virus (JEV, Nakayama strain) stocks were generated by infecting BHK-21 cells at a MOI of 0.01 and collecting clarified supernatants after centrifugation. Rotavirus (Wa strain) stocks were prepared by infecting MA104 cells at a MOI of 0.01. All virus stocks were aliquoted and stored at –80°C until use.

### *In vitro* Cytotoxicity and Antioxidative Activity Tests

The *in vitro* cytotoxicity of lutein was evaluated using the Cell Counting Kit-8 (CCK-8) assay (Dojindo Laboratories, Japan) and the MTT assay (Sigma-Aldrich). MDCK cells were treated with lutein at various concentrations and incubated at 37°C for various time points (1–72 h). For the CCK-8 assay, 10 μl of the CCK-8 reagent was added to each well, followed by incubation at 37°C for 1 h. The absorbance was measured at 450 nm. For the MTT assay, 10 μl of the MTT reagent was added to the cells and incubated at 37°C for 3 h. Subsequently, 100 μl of DMSO was added to each well, and the plates were incubated at room temperature for 30 min, and absorbance was measured at 570 nm. The antioxidant activity of lutein was assessed using the 2,2-diphenyl-1-picrylhydrazyl (DPPH) radical-scavenging assay (Dojindo Laboratories). 20 μl lutein at various concentrations was mixed with 80 μl assay buffer and 100 μl of DPPH solution, followed by incubation for 30 min. The absorbance was measured at 517 nm.

### Viral Plaque Assays for Influenza Viruses, JEV, and Rotavirus Titration

Viral plaque assays were performed to determine the titers of influenza viruses, JEV, and rotavirus. For influenza viruses, ten-fold serial dilutions of virus samples were inoculated onto confluent MDCK cells and incubated for 45 min to allow virus adsorption. After removing the inocula, cells were washed and overlaid with DMEM containing 1% low-melting agarose (Lonza, USA), 2.5 μg/ml trypsin (Gibco), and antibiotic–antimycotic solution (GenDEPOT, Republic of Korea), and incubated at 37°C in a 5% CO_2_ incubator for 2–3 days until plaques appeared. JEV titers were determined in BHK-21 cells. Ten-fold serial dilutions of virus samples prepared in DMEM containing 2% FBS were added to the cells and incubated for 2 h on a shaking incubator for viral adsorption. Cells were washed and overlaid with EMEM (Quality Biologicals, USA) containing 0.9% low-melting agarose and 8% FBS, and incubated at 37°C in 5% CO_2_ incubator for 3 days until plaques became visible. Rotavirus plaque assays were conducted in MA104 cells. Prior to infection, virus was activated with 10 μg/ml trypsin for 30 min at 37°C, serially diluted, and added to the cell monolayers. After 1 h of virus adsorption at 37°C, the inocula were removed, and cells were washed and overlaid with MEM containing 1% low-melting agarose, 2% FBS, and 1 μg/ml trypsin. Plates were incubated at 37°C in a 5% CO_2_ incubator for 3–5 days until plaques developed. For plaque visualization, cells were fixed with 4% paraformaldehyde (Biosesang), overlays were removed, and plaques were stained with 1% crystal violet solution (Sigma-Aldrich).

### Influenza Virus Attachment Inhibition Assay

To evaluate the inhibitory effect of lutein on influenza virus attachment to host cells, an attachment inhibition assay was performed with minor modifications to a previously described method [31]. MDCK cell monolayers cultured in 6-well plates were pre-chilled at 4°C for 10–20 min. Approximately 100 PFUs of virus were mixed with various concentrations of lutein and added to the pre-chilled cells, followed by incubation at 4°C for 45 min to allow viral attachment to the cell surface without virus internalization. After the attachment period, cells were washed with cold PBS to remove unbound virus. Subsequently, 3 mL of overlay medium was added to each well, and the cells were incubated at 37°C in a 5% CO_2_ incubator for 2–3 days to allow plaque formation. Once plaques had formed, cells were fixed with 4% formaldehyde. After fixation, the overlay medium was removed, and the cells were stained with crystal violet solution to visualize plaques.

### Virus-Coating ELISA for Assessing Lutein Binding to Influenza Viral HA

To assess whether lutein specifically binds to the influenza HA protein, a virus-coating ELISA was performed. 96–well immunoplates were coated with A/PR8 virus at a concentration of 10^4^ PFU/well and incubated at 4°C for 18 h. Plates were then washed three times with PBST and blocked with 1% BSA in PBS for 1 h at 37°C. After washing, two-fold serial dilutions of lutein were added and incubated at 37°C for 2 h. Following another wash, monoclonal anti-influenza A/H1N1 virus HA broadly reactive antibody (AcroBiosystems, clone 5B2) was diluted to 10 ng/ml and added to each well (100 μl/well). Subsequently, HRP-conjugated goat anti-human IgG Fc secondary antibody (100 μl/well) was applied after washing. After additional washing steps, 50 μl of TMB substrate was added and incubated at 37°C for 20 min. The reaction was terminated with 50 μl of 2 N H_2_SO_4_, and absorbance was measured at 450 nm using a microplate reader.

### Hemagglutination Inhibition (HI) Assay

The HI assay was performed to determine the HA units (HAU) of IAVs. In V-bottom 96-well plates, 50 μl of two-serial dilutions of influenza viruses were mixed with an equal volume of 1% chicken red blood cells (cRBCs, Innovative Research, USA). The mixture was incubated at 4°C for 1 h to allow hemagglutination. The HA titers were determined as the highest dilution of the virus that resulted in complete hemagglutination. The HI assay was conducted to assess the potential of lutein to inhibit virus-induced hemagglutination. In V-bottom 96-well plates, 25 μl of two-fold serial dilutions of lutein was incubated with 4 HAU of IAVs (25 μl) at 37°C for 30 min or 2 h. Following incubation, 50 μl of 1% cRBCs was added to each well, and the plate was further incubated at 4°C for 1 h. The inhibition of hemagglutination was then evaluated.

### NA Inhibition Assay by Lectin-Based ELISA and the MUNANA Assay

The NA enzyme activity of IAVs was evaluated using a lectin-based enzyme-linked assay. In this assay, viral NA cleaves terminal sialic acids from fetuin, exposing galactose residues that are specifically recognized by lectin, thereby allowing colorimetric detection of NA activity. Briefly, 96-well plates were coated with 100 μl/well of fetuin (Sigma-Aldrich) and incubated at 4°C for 18 h. Two-fold serial dilutions of influenza viruses were added to the fetuin-coated wells and incubated at 37°C for 1 h. After washing, 100 μl of HRP-conjugated peanut lectin (Sigma-Aldrich) was added to each well and incubated for 1 h at room temperature. Plates were washed, and 100 μl of TMB substrate (Thermo Scientific, USA) was added, followed by incubation for 5 min. The reaction was terminated with 50 μl/well of 2 N H_2_SO_4_, and absorbance was measured at 450 nm. For the NI assay, two-fold serial dilutions of lutein or OP were mixed with influenza viruses at titers corresponding to an NA activity of OD_450_ = 1. The mixtures were incubated at 37°C for 30 min or 2 h before being transferred to fetuin-coated plates, and NA activity was determined as described above. In parallel, NA inhibition activity of lutein was also assessed using the fluorogenic substrate 2'-(4-methylumbelliferyl)-α-D-N-acetylneuraminic acid (MUNANA) (Sigma-Aldrich). Influenza viruses (25 μl/well) were mixed with serial concentrations of lutein (25 μl/well) in 96-well black flat-bottom plates and incubated at 37°C for 30 min or 2 h. Subsequently, 50 μl of MUNANA substrate (200 μM in 33.3 mM MES buffer, 4 mM CaCl_2_, pH 6.5) was added, and the reaction was allowed to proceed at 37°C for 1 h. The enzymatic reaction was stopped with 100 μl of stop solution (0.2 M Na_2_CO_3_), and fluorescence was measured using a microplate reader (excitation 355 nm, emission 450 nm). NA inhibition was calculated relative to virus-only controls, with OP serving as a positive control.

### Time-of-Addition (TOA) Experiments

TOA assay was performed to determine the specific stages at which lutein exerts its antiviral activity. TOA assay was conducted under three different conditions, wherein lutein was added to cells or viruses at different time points. 1) Pre-treatment to virus: To evaluate the virucidal activity of lutein, influenza viruses were incubated with various concentrations of lutein at 37°C for 30 min or 2 h. The mixtures were then inoculated onto MDCK cells to assess viral infectivity. 2) Co-treatment: To investigate whether lutein inhibits viral entry, influenza viruses were mixed with various concentrations of lutein and immediately applied to MDCK cells for viral infection. 3) Post-treatment: To examine whether lutein affects the viral release stage, MDCK cells were first infected with influenza viruses and subsequently treated with various concentrations of lutein. In all conditions, MDCK cells were infected with influenza viruses at a MOI of 0.01 (100 PFU). Cell viability was assessed using the MTT assay at 48 h post-infection (hpi) for A/MA81 and 72 hpi for A/PR8.

### GFP Fluorescence Analysis

To investigate the antiviral activity of lutein quantitatively, fluorescence imaging analysis was performed using the A/PR8-GFP reporter virus that encodes GFP in the NS segment [30]. MDCK cells grown in 96-well plates were infected with the A/PR8-GFP reporter virus at MOI of 1. At 16 hpi, the culture medium was removed, washed with PBS, and 100 μl/well of Hoechst 33342 (Thermo Scientific) was added to the wells for 10 min to stain the nuclei of the cells. The cells were washed with PBS and fluorescence imaging analysis was performed using the Cytation 1 fluorescence microscope (BioTeK, USA).

### Dynamic Light Scattering (DLS) Analysis

The particle size distribution of influenza virions was analyzed using DLS. Briefly, purified virus suspensions were treated with 50 μM lutein and incubated at 37°C for 2 h. Following treatment, virus samples were clarified by low-speed centrifugation to remove aggregates and then diluted in PBS to a final volume of 1 ml. The hydrodynamic diameter and polydispersity index (PDI) of viral particles were measured using a Zetasizer Nano ZS instrument (Malvern Instruments, UK) equipped with a 633 nm He–Ne laser at a scattering angle of 173°. All measurements were performed at 25°C, and each sample was analyzed in triplicate. Data were processed with Zetasizer software (version 3.3.1.5, ZS xplorer software).

### Quantitative Real-Time PCR (qRT-PCR)

MDCK cells were infected with A/PR8 at a MOI of 0.01 (10^4^ PFU). Following infection, the cells were treated with lutein at final concentrations of 50 μM and 100 μM. At 24 hpi, total RNA was extracted using TRIzol reagent (Thermo Scientific) according to the manufacturer’s instructions. The concentration of the extracted RNA was assessed using a UV spectrophotometer. A total of 2 μg of RNA was reverse transcribed into complementary DNA using the High-Capacity cDNA Reverse Transcription Kit (Applied Biosystems, USA). qRT-PCR was performed using the QuantStudio 1 system (Thermo Scientific) under the following thermal cycling conditions: initial denaturation at 95°C for 10 min, followed by 40 cycles of denaturation at 95°C for 15 sec and annealing/extension at 60°C for 1 min. The relative expression levels of influenza virus PB1, NP, and M1 mRNA were determined using the 2^-ΔΔCt^ method, with GAPDH as the internal control. The primer sequences used for qRT-PCR are listed in Table S1.

### Statistical Analysis

All experiments were conducted at least three times, and data are presented as the mean ± standard error of the mean (SEM). Statistical comparisons between two groups were performed using Student’s *t*-test. A *P*-value less than 0.05 was considered statistically significant. The significance levels were denoted as follows: ****; *P* < 0.0001, ***; *P* < 0.001, **; *P* < 0.01, and *; *P* < 0.05. Nonlinear regression analysis was conducted using GraphPad Prism 9.0 software.

## Results

### *In Vitro* Cytotoxicity and Antioxidative Activity of Lutein

**Fig. 1:**
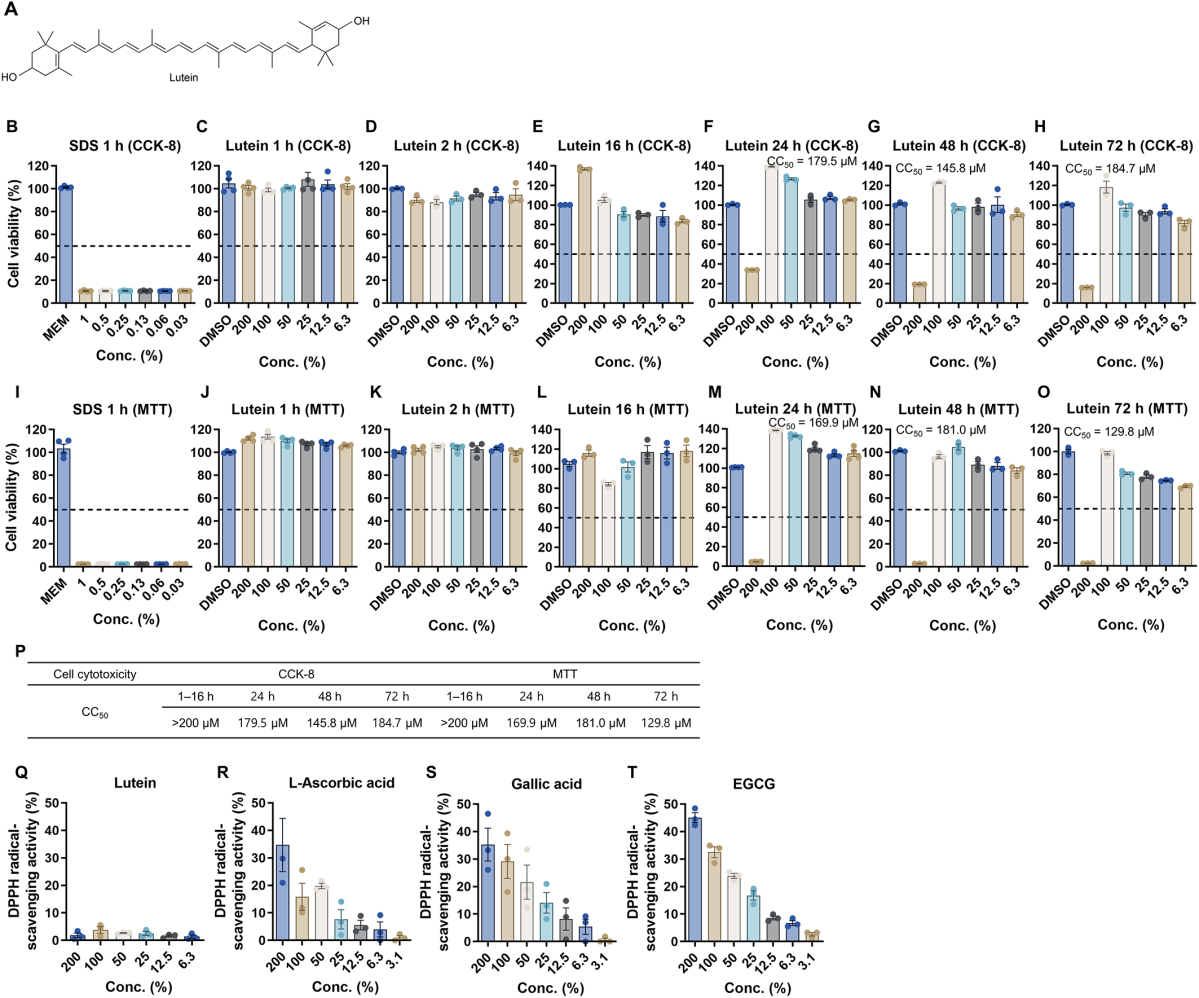
*In vitro* cytotoxicity and antioxidative activity of lutein. (**A**) Chemical structure of lutein. (**B–P**) *In vitro* cytotoxicity of lutein. MDCK cells were treated with various concentrations of lutein for 1–72 h at 37°C. As a positive control, SDS were treated to MDCK cells for 1 h. Cell viability was measured using CCK-8 (**B–H**) and MTT assay (**I–O**). DMSO was used as the vehicle control, and the dotted line represents 50% cell viability. The CC_50_ values of cytotoxicity are summarized in P. (**Q–T**) DPPH radical-scavenging activity of lutein (**Q**), L-ascorbic acid (**R**), gallic acid (**S**), and EGCG (**T**).

Lutein consists of a nonpolar hydrocarbon chain with cyclohexene rings at both ends, each bearing hydroxyl groups (Fig. 1A). To evaluate *in vitro* cytotoxicity, MDCK cells were treated with various concentrations of lutein for 1–72 h at 37°C, and cell viability was measured by CCK-8 and MTT assays, using sodium dodecyl sulfate (SDS) as a positive control. In CCK-8 assay, SDS induced high level of cytotoxicity at 0.031–1% after 1 h treatment (Fig. 1B). In contrast, lutein showed no cytotoxicity in CCK-8 assay for 1–16 h treatment (Fig. 1C–1E). However, prolonged exposure (24–72 h) caused cytotoxicity at 200 μM, while lower concentrations remained non-cytotoxic (Fig. 1F–1H). In MTT assay, SDS showed cytotoxicity after 1 h treatment (Fig. 1I), but lutein displayed cytotoxicity only after 24–72 h treatment at 200 μM (Fig. 1J–1O). The CC_50_ values of the cytotoxicity tests are summarized in Fig. 1P. Based on these results, subsequent antiviral assays with lutein were performed at the non-cytotoxic concentrations. The antioxidative activity of lutein was assessed using the DPPH assay. Lutein exhibited minimal radical-scavenging activity across 6.3–200 μM (Fig. 1Q), whereas standard antioxidants, L-ascorbic acid, gallic acid, and EGCG, showed 34.7%, 35.3%, and 45.1% radical-scavenging activity at 200 μM, respectively (Fig. 1R–1T), showing low antioxidative activity of lutein.

### Virucidal Activity of Lutein against Enveloped and Non-Enveloped Viruses

**Fig. 2:**
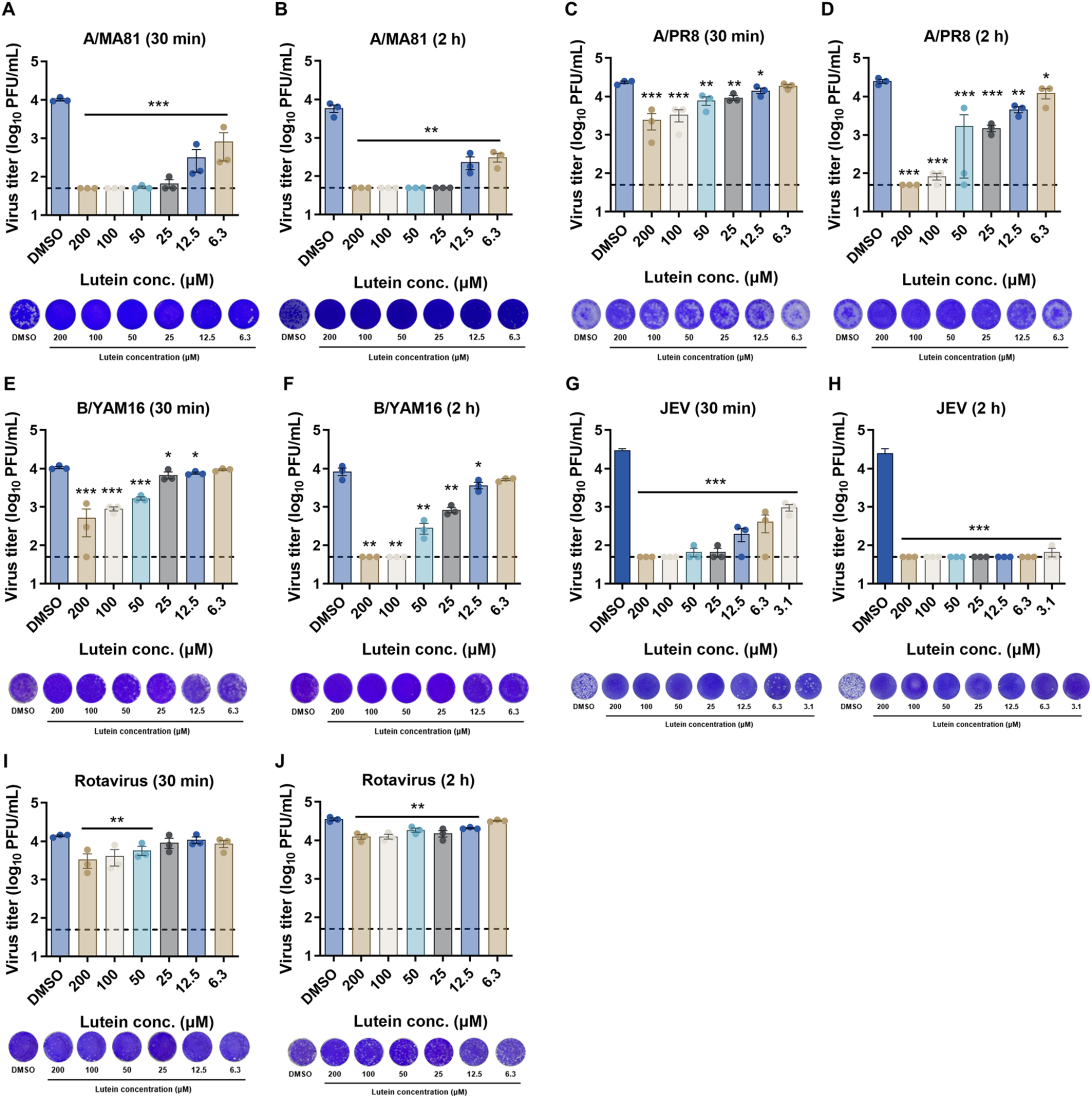
Virucidal activity of lutein against enveloped and non-enveloped viruses. (**A–F**) Virucidal activity of lutein against IAVs and IBV. Various concentrations of lutein were treated to 10^4^ PFU of each virus and incubated at 37°C for 30 min or 2 h at 37°C, and the residual viral titers were measured by viral plaque assay. Residual viral titers of A/MA81 (**A** and **B**), A/PR8 (**C** and **D**), and B/YAM16 (**E** and **F**). (**G** and **H**) Virucidal activity of lutein against JEV. (I and J) Virucidal activity of lutein against rotavirus. Representative images of plaque assays of a 10^-1^ dilution are shown. DMSO was used as the vehicle control, and the dotted line indicates the detection limit of 1.699.

To assess the virucidal activity of lutein against IAVs, 6.3–200 μM lutein was incubated with 10^4^ PFU of A/MA81 or A/PR8 for 30 min or 2 h at 37°C, followed by viral plaque assay for residual viral titration. For A/MA81, 30-min incubation completely inactivated the virus at 100–200 μM, with significant reductions observed at 6.3–50 μM (Fig. 2A). Extending incubation to 2 h enhanced activity, achieving complete inactivation at 25–200 μM (Fig. 2B). For A/PR8, 30-min treatment with 100–200 μM lutein reduced titers by 9.5-fold but did not achieve complete inactivation (Fig. 2C). After 2 h-treatment, complete inactivation occurred at 200 μM, with significant reductions at 6.3–100 μM (Fig. 2D). The virucidal activity of lutein was also tested against enveloped viruses including IBV and JEV, and non-enveloped rotavirus. Lutein showed time-dependent inactivation of IBV (Fig. 2E and 2F) and displayed stronger activity against JEV, completely inactivating it at 6.3 μM by 2 h-treatment (Fig. 2G and 2H). In contrast, virucidal activity against non-enveloped rotavirus was relatively weaker (<1-log reduction) even for 2 h treatment (Fig. 2I and 2J). These results demonstrate that lutein exerts potent virucidal activity against enveloped viruses including influenza viruses and JEV, but limited effects on a non-enveloped virus.

### Virucidal Activity of Lutein Assessed by CPE Reduction and GFP Analysis

**Fig. 3:**
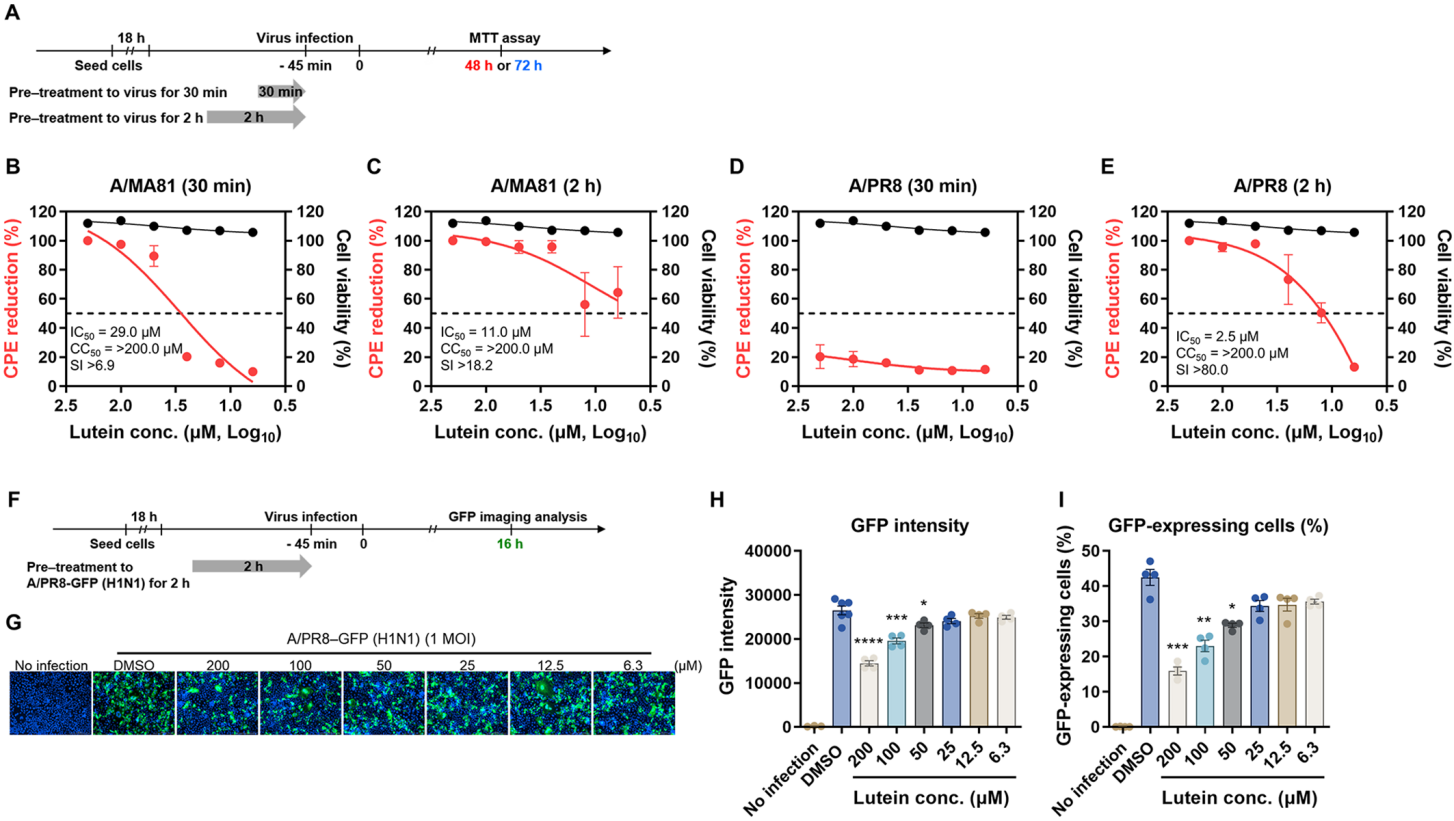
Virucidal activity of lutein confirmed by CPE reduction assay and GFP analysis. (**A**) Schematic of CPE reduction assay of pre-treatment to virus experiments. (**B–E**) CPE reduction assay after lutein treatment to the virus. 100 PFU of the virus was treated with lutein and incubated for 30 min or 2 h at 37°C. After the incubation, lutein-virus mixtures were added to MDCK cells for viral infection. CPE reduction of the cells infected with A/MA81 (**B** and **C**) and A/PR8 (**D** and **E**). Cell viability determined by MTT assay after lutein treatment to MDCK cells for 1 h are also plotted in graphs. SI of each assay was determined using the ratio of CC_50_ to IC_50_, CC_50_/IC_50_ (*n* = 3 or 4). (**F–H**) Fluorescence analysis using a A/PR8-GFP reporter virus. Two-fold serial dilutions of lutein were incubated with 100 PFU of A/PR8-GFP reporter virus, and the mixtures were added to MDCK cells. After 16 h, nuclei were stained with Hoechst 33342, and fluorescence images were acquired using a fluorescence microscope (**F**). GFP image (**G**), GFP intensity (**H**), and GFP-expressing cell (%) (**I**) are shown.

The virucidal activity of lutein against IAV was further evaluated using a cytopathic effect (CPE) reduction assay and the A/PR8-GFP reporter virus. A/MA81 and A/PR8 were pre-treated with lutein at 37°C for 30 min or 2 h before infection, and CPE was measured via the MTT assay at 48 hpi for A/MA81 and 72 hpi for A/PR8 (Fig. 3A). For A/MA81, 30-min pre-treatment to virus with 6.3–200 μM lutein reduced CPE by 10–100%, with the IC_50_ of 29.0 μM (Fig. 3B). Extending pre-treatment to 2 h enhanced the activity, lowering the IC_50_ to 11.0 μM (Fig. 3C). For A/PR8, 30-min pre-treatment to virus had minimal effect (11.2–20.3%), with IC_50_ not determinable (Fig. 3D). However, 2 h pre-treatment to virus markedly reduced CPE: 95.5–100% at 50–200 μM, 73.3% at 25 μM, and 50.4% at 12.5 μM (IC_50_ = 2.5 μM) (Fig. 3E). The selective index (SI) was >6.9, >18.2, and >80, except for 30-min pre-treatment to A/PR8. Fluorescence imaging using A/PR8-GFP further validated these findings (Fig. 3F). After 2 h pre-treatment with lutein, viral infection of MDCK cells resulted in a marked reduction of GFP expression at 50–200 μM compared to DMSO control (Fig. 3G). GFP intensity and GFP-positive cell proportion significantly decreased by pre-treatment of lutein to virus at 50–200 μM (Fig. 3H and 3I). These results indicate that lutein pre-treatment to virus reduces IAV-induced CPE in a concentration- and time-dependent manner, and high lutein concentrations suppress viral GFP expression, confirming its virucidal activity.

### Lutein Inhibits Influenza Viral Entry via HA-Mediated Receptor Binding

**Fig. 4:**
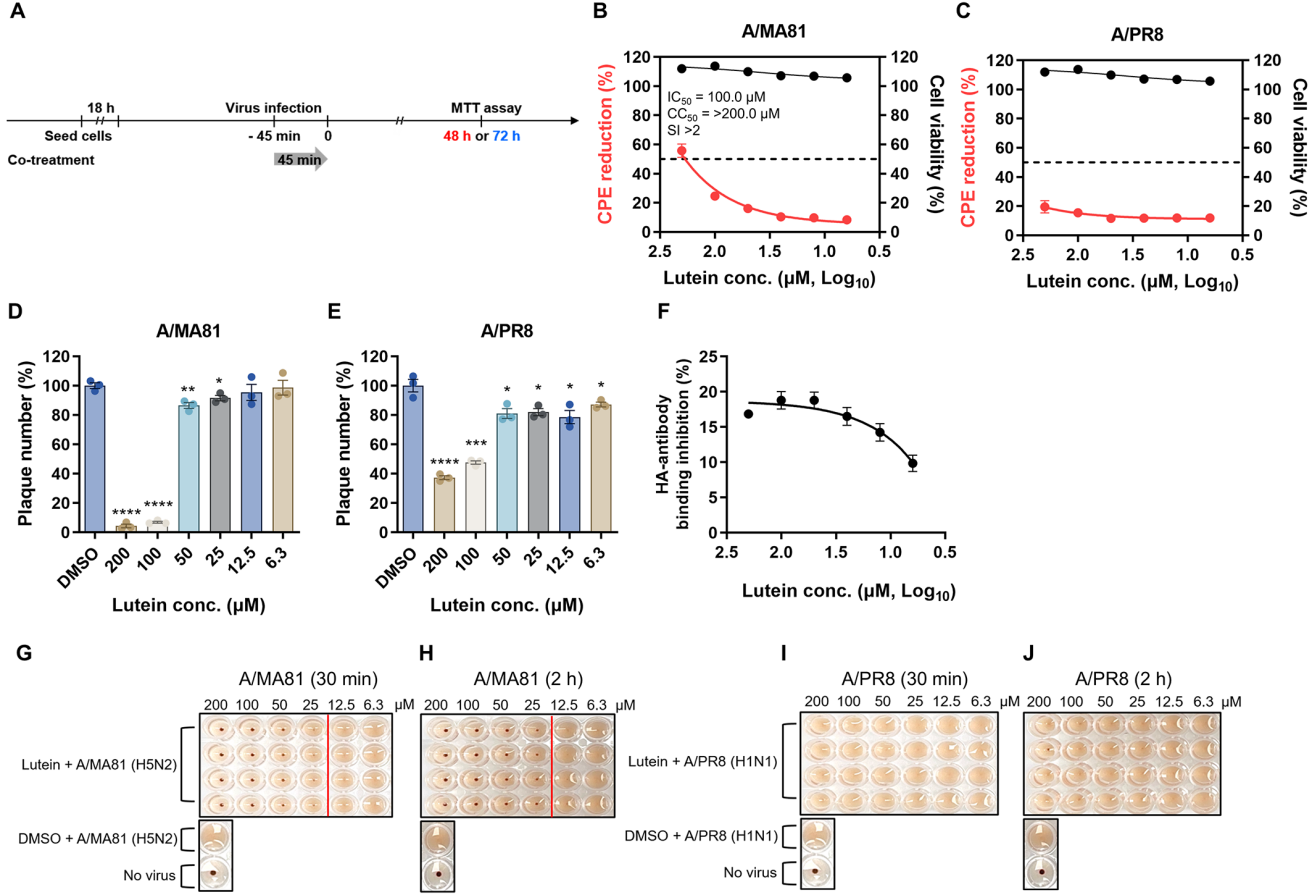
Inhibition of viral entry and HA activity by lutein. (**A**) Schematic representation of the CPE reduction assay of co-treatment experiments. (**B** and **C**) CPE reduction following co-treatment of influenza virus and lutein. MDCK cells were infected with 100 PFU of influenza virus pre-incubated with various concentrations of lutein. CPE reduction was assessed at 48 hpi with A/MA81 (**B**) and at 72 hpi with A/PR8 (**C**). Cell viability was determined by MTT assay after 1 h exposure of MDCK cells to lutein. The SI values were calculated as the ratio of CC_50_ to IC_50_ (CC_50_/IC_50_) (*n* = 3 or 4). (**D** and **E**) Virus attachment inhibition by lutein. Pre-chilled MDCK cells were co-treated with 100 PFU of influenza virus and lutein, followed by incubation at 4°C for 1 h. After washing, residual virus infectivity was quantified by plaque assay. (**F**) Virus-coating ELISA. Virus-coating ELISA was performed to assess the effect of lutein on HA–antibody interaction. HA binding inhibition was expressed as the relative OD_450nm_ to DMSO-treated control. (**G–J**) HI activity of lutein against influenza virus. 4 HA units of IAVs were incubated with lutein for 30 min or 2 h at 37°C, followed by the addition of cRBCs and incubation at 4°C for 1 h to allow hemagglutination. HI assay results from four replicates are shown for A/MA81 (**G** and **H**) and A/PR8 (**I** and **J**). DMSO was used as the vehicle control.

To evaluate whether lutein interferes with viral entry, A/MA81 and A/PR8 viruses were co-treated with lutein and immediately inoculated onto MDCK cells, followed by measurement of CPE reduction using the MTT assay (Fig. 4A). For A/MA81, lutein reduced CPE by 8.4–55.7% at 6.3–200 μM (IC_50_ = 100.0 μM) (Fig. 4B), whereas A/PR8 exhibited only modest reduction of 11.6–19.5%, with no determinable IC_50_ (Fig. 4C). In the virus attachment inhibition assay, lutein markedly decreased plaque formation of A/MA81 to 4.4%, 6.9%, and 86.5% at 200, 100, and 50 μM, respectively (Fig. 4D). For A/PR8, plaque numbers were reduced in a dose-dependent manner to 37.2–47.7% (Fig. 4E), indicating that lutein inhibits viral attachment to host cells. To further examine whether lutein directly binds to influenza viral HA, a virus-coating ELISA was performed. Pre-treatment of A/PR8 with lutein for 2 h at 37°C resulted in inhibition of HA–antibody binding by 9.9–18.8% at 6.3–200 μM (Fig. 4F), suggesting weak but dose-dependent interactions between lutein and viral HA. Furthermore, a HI assay was conducted to assess whether lutein interferes with HA-mediated receptor binding. Lutein blocked the receptor binding activity of A/MA81 HA at 25–200 μM, regardless of incubation time (Fig. 4G and 4H), whereas no HI activity was observed for A/PR8 (Fig. 4I and 4L). Together, these findings indicate that lutein inhibits influenza viral entry by interfering with attachment and HA-mediated receptor binding, likely through direct interaction with viral HA. Lutein exhibited stronger antiviral activity against A/MA81 than against A/PR8, as demonstrated by CPE reduction, virus attachment inhibition, and HI assays.

### Post-Treatment of Lutein Inhibits Influenza Viral Replication

**Fig. 5:**
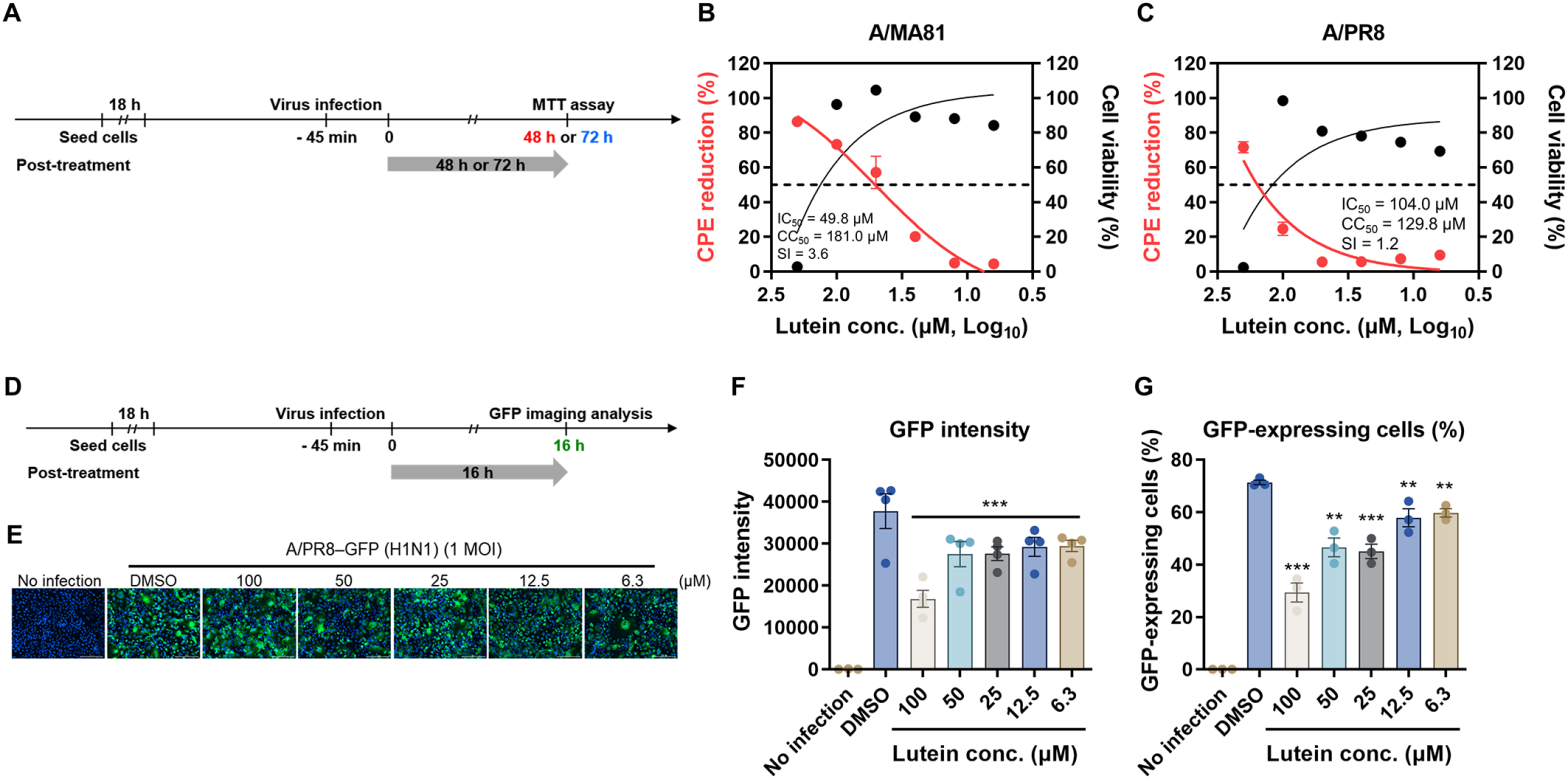
Inhibition of influenza viral release by lutein. (**A**) Schematic of the CPE reduction assay of post-treatment experiments. (**B** and **C**) CPE reduction after post-treatment of lutein to the virus-infected cells. MDCK cells were infected with 100 PFU of influenza virus, followed by treatment of lutein. CPE reduction after infection with A/MA81 (**B**) was measured at 48 hpi, while CPE reduction after infection with A/PR8 (**C**) was measured at 72 hpi. Cell viability determined by MTT assay after lutein treatment to MDCK cells for 48 or 72 h are also plotted in graphs (*n* = 3 or 4). (**D–F**) Fluorescence analysis using a A/PR8-GFP reporter virus. MDCK cells were infected with 10^4^ PFU of the virus, and the cells were treated with lutein. At 16 hpi, the cells were stained with Hoechst 33342 and analyzed by fluorescence imaging (**D**). GFP image (**E**), GFP intensity (**F**), and GFP-expressing cell (%) (**G**) are shown.

To examine whether lutein affects the whole viral life cycle, MDCK cells were infected with A/MA81 or A/PR8, followed by post-treatment with lutein, and CPE was assessed at 48 hpi or 72 hpi using the MTT assay (Fig. 5A). In A/MA81-infected cells, lutein reduced CPE by 4.3–73.2% at concentrations over CC_50_, with the IC_50_ of 49.8 μM (Fig. 5B). For A/PR8-infected cells, post-treatment led to CPE reduction of 5.2–24.6% at concentrations over CC_50_, with the IC_50_ of 104.0 μM (Fig. 5C). The SI values of the assay were calculated as 3.6 and 1.2 for A/MA81 and A/PR8 viruses, respectively. Fluorescence imaging using the A/PR8-GFP virus further confirmed the antiviral effects during post-treatment (Fig. 5D). Lutein post-treatment caused dose-dependent reductions in GFP expression compared to controls (Fig. 5E). Quantitative analysis showed 2.4-fold reduction at 100 μM and 1.3–1.4-fold reduction at 6.3–50 μM lutein in GFP intensity (Fig. 5F). Similarly, the proportion of GFP-positive cells decreased 2.4-fold at 100 μM and 1.2–1.6-fold at 6.3–50 μM (Fig. 5G). In summary, post-treatment with lutein reduced CPE and GFP expression in virus-infected cells, with stronger effects against A/MA81 than A/PR8. These results suggest that post-treatment of lutein inhibits the viral replication.

### NA Inhibition Activity of Lutein

**Fig. 6:**
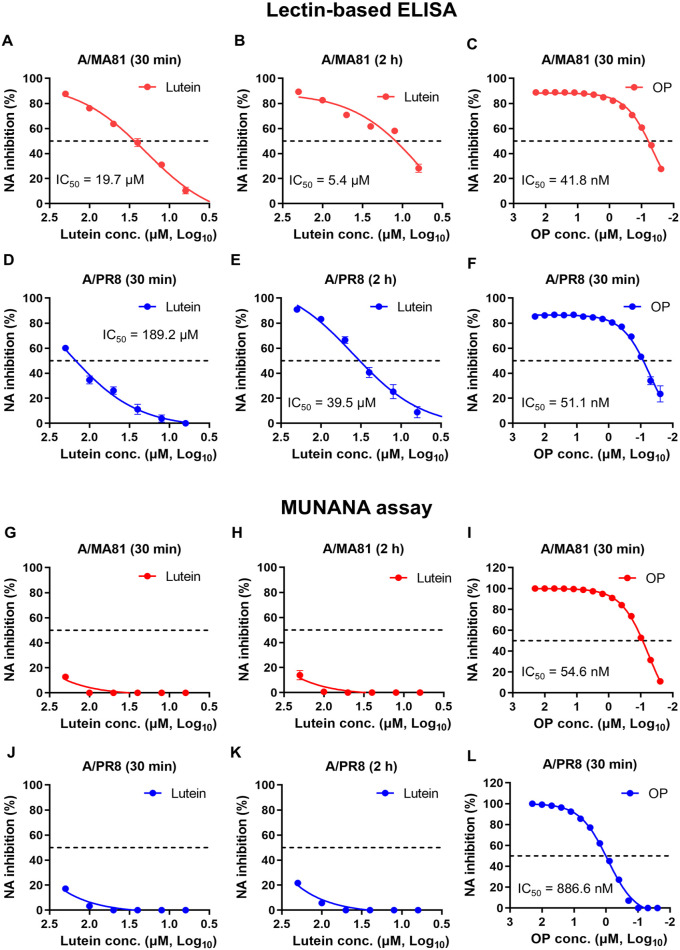
NA inhibition of lutein assessed by lectin-based ELISA and the MUNANA assay. (**A–F**) Lectin-based ELISA results. Two-fold serial dilutions of lutein or OP were treated to influenza virus for 30 min or 2 h at 37°C. NA inhibition activity was measured by a lectin-based ELISA method. NA inhibition of lutein or OP against A/MA81 (**A–C**) and against A/ PR8 (**D–F**). (**G–L**) The MUNANA assay results. NA inhibition of lutein or OP against A/MA81 (**G–I**) and against A/PR8 (**J–L**). IC_50_ values are shown in each graph (*n* = 3 or 4).

Influenza virus NA cleaves sialic acid from host receptors, facilitating virion release. To assess whether lutein inhibits NA activity, two assays—lectin-based ELISA and MUNANA assay—were performed using A/MA81 and A/PR8, using OP as a positive control. In the lectin-based ELISA, lutein treatment of A/MA81 for 30 min inhibited NA activity by 10.5–87.6% at 6.3–200 μM (IC_50_ = 19.7 μM) (Fig. 6A). Extending incubation to 2 h enhanced NA inhibition (28.3–89.3%) and decreased the IC_50_ to 5.4 μM (Fig. 6B). OP showed strong NA inhibition against A/MA81 at nanomolar concentrations (IC_50_ = 41.8 nM) (Fig. 6C). For A/PR8, 30-min treatment produced 0.8–60.1% inhibition at 6.3–200 μM (IC_50_ = 189.2 μM) (Fig. 6D), while 2 h treatment increased NA inhibition to 8.9–90.9%, lowering the IC_50_ to 39.5 μM (Fig. 6E). OP also showed strong NA inhibition against A/PR8 (IC_50_ = 51.1 nM) (Fig. 6F). Overall, lutein exhibited dose- and time-dependent NA inhibition activity, exerting more pronounced activity against A/MA81 than A/PR8. It has been reported that HA-inhibiting compounds can non-specifically reduce NA activity through steric hindrance [32, 33]. To determine whether lutein inhibits NA by directly targeting the catalytic site of the NA, a MUNANA assay measuring the hydrolysis of a small fluorogenic substrate was performed. The MUNANA assay showed only modest NA inhibition by lutein for A/MA81 regardless of incubation time (Fig. 6G and 6H), whereas OP strongly inhibited the NA activity of the virus (IC_50_ = 54.6 nM)(Fig. 6I). Similarly, lutein rarely inhibited the NA activity of A/PR8 (Fig. 6J and 6K), while OP showed strong NA inhibition activity against the virus, with the IC_50_ of 886.6 nM (Fig. 6L). The discrepancy between the two assays likely reflects their different principles: the lectin-based ELISA detects NA cleavage of glycoprotein-linked sialic acids and may reflect steric or conformational interference by lutein on the virion surface, whereas the MUNANA assay reflects direct active-site inhibition. These results suggest that lutein inhibits NA activity primarily by affecting substrate accessibility or glycoprotein interactions rather than directly binding the catalytic site.

### Lutein Disrupts the Physical Integrity of Influenza Virus Particles

**Fig. 7:**
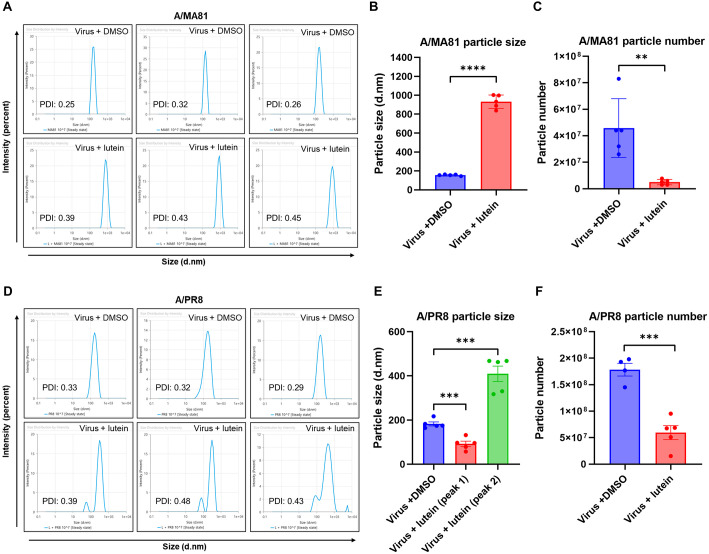
Effect of lutein on the size distribution and particle number of influenza viruses. DLS analysis of influenza virus particles following lutein treatment. A/MA81 or A/PR8 suspensions containing 10^8^ PFU/mL were incubated with DMSO or 50 μM lutein for 2 h at 37°C. After incubation, the mixtures were subjected to DLS measurement to determine particle size distribution and concentration. The histograms show the average hydrodynamic diameter and PDI of virus particles (**A** and **D**). Particle size (**B** and **E**) and particle number (**C** and **F**) between DMSO- and lutein-treated groups (*n* = 4 or 5).

DLS analysis was performed to evaluate the effects of lutein on influenza virus particle integrity. For A/MA81, DMSO-treated controls displayed a homogeneous population with an average hydrodynamic diameter of 161–164 nm and low polydispersity (PDI: 0.25–0.32) (Fig. 7A). In contrast, lutein treatment markedly increased particle size to 838–932 nm and elevated PDI values (0.39–0.45), indicating particle aggregation and reduced uniformity. While DMSO-treated virus exhibited an average particle size of 156.2 nm, lutein treatment significantly increased the size to 931.8 nm, representing a 6-fold enlargement (Fig. 7B). Moreover, lutein-treated virus particles showed a 9.2-fold reduction in particle number compared with the control (Fig. 7C). Similarly, DMSO-treated A/PR8 displayed sizes of 164–175 nm with PDI <0.34, whereas lutein treatment resulted in a heterogeneous, multimodal distribution with peaks at 57–96 nm, 317–463 nm, and 5,300 nm (PDI: 0.39–0.48)(Fig. 7D), suggesting severe disruption of particle integrity. Together, these results indicate that lutein destabilizes the structural integrity of influenza virus particles, leading to aggregation, heterogeneity, and a marked reduction in intact virions. Of note, the DLS profiles revealed strain-specific differences: A/MA81 primarily exhibited particle enlargement and reduced counts, whereas A/PR8 showed more pronounced heterogeneity and fragmentation, indicating differential susceptibility to lutein.

### Lutein Inhibits Influenza Viral Growth and Viral mRNA Expression

**Fig. 8:**
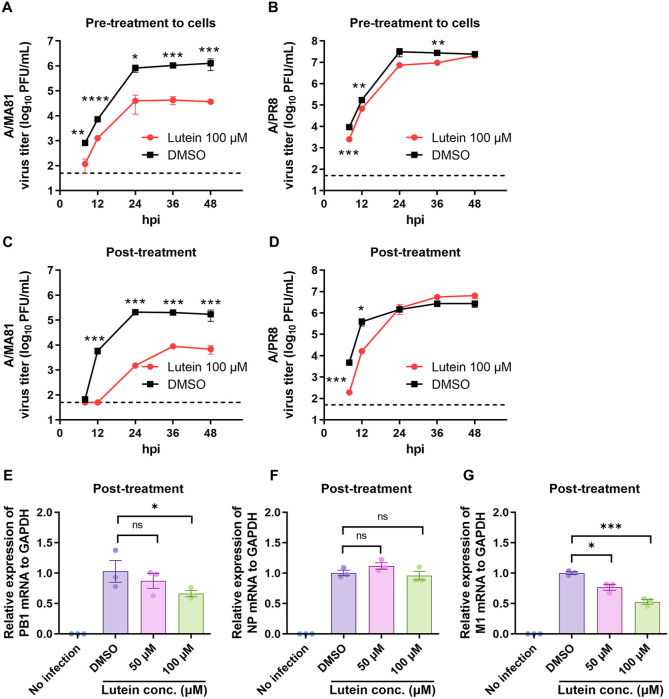
Inhibition of viral growth kinetics and mRNA expression by lutein. (**A** and **B**) Effects of lutein pre-treatment to cells on viral growth kinetics of influenza viruses. 100 μM lutein was added to MDCK cells for 24 h at 37°C, and the cells were infected with 0.01 MOI of influenza virus. The viral titers in the supernatants harvested at different time points were measured by a plaque assay. The viral titers of A/MA81 (**A**) and A/PR8 (**B**). (**C** and **D**) Effects of lutein post-treatment on viral growth kinetics of influenza viruses. MDCK cells were infected with 0.01 MOI of influenza virus and 100 μM lutein was added to the cells. The viral titers in the supernatants harvested at different time points were measured by a plaque assay. The viral titers of A/ MA81 (**C**) and A/PR8 (**D**). The dotted line represents the detection limit, 1.699 (*n* = 3). (**E–G**) qRT-PCR results. MDCK cells were infected with 0.01 MOI of A/PR8, followed by post-treatment with 50 μM or 100 μM lutein. At 24 hpi, mRNA expression levels of PB1 (**E**), NP (**F**), and M1 (**G**) were analyzed by qRT-PCR. DMSO was used as the controls.

To evaluate the effect of lutein on IAV replication, viral growth kinetics were analyzed in MDCK cells with pre-or post-treatment to cells of lutein, and viral titers were measured by plaque assay. For A/MA81, pre-treatment to cells with 100 μM lutein reduced titers from 8.2 × 10^5^ PFU/ml to 4.0 × 10^4^ PFU/ml at 24 hpi, a 20.5-fold decrease compared to DMSO control (Fig. 8A). For A/PR8, pre-treatment to cells of lutein decreased titers from 3.1 × 10^7^ PFU/ml to 7.3 × 10^6^ PFU/ml, a 4.2-fold reduction (Fig. 8B). Post-treatment to cells with 100 μM lutein also inhibited viral replication. In A/MA81-infected cells, viral growth was almost completely suppressed at 8–12 hpi, with titers maintained at 1.5–8.9 × 10^3^ PFU/ml during 24–48 hpi, representing >100-fold reduction compared to controls (Fig. 8C). For A/PR8, post-treatment to cells delayed early replication, with titers of 1.9 × 10² PFU/ml at 8 hpi and 1.6 × 10^4^ PFU/ml at 12 hpi (24.4-fold reduction), although titers approached control levels by 24 hpi (Fig. 8D). These results indicate that lutein inhibits IAV replication more effectively for A/MA81 than A/PR8, especially at early time points. To assess effects on viral mRNA, A/PR8-infected cells were post-treated to cells with 50 or 100 μM lutein. PB1 mRNA was reduced by 12.7% and 33.7%, respectively (Fig. 8E), while NP mRNA remained largely unchanged (Fig. 8F). M1 mRNA expression was significantly decreased, with 50 μM lutein reducing levels by 23.4% and 100 μM by 47.2% (Fig. 8G). Overall, lutein suppresses IAV replication by reducing viral titers and downregulating viral mRNA expression, with stronger effects observed when applied pre-infection or at early stages post-infection.

## Discussion

In this study, we extensively investigated the antiviral potential of lutein against IAVs, through cellular, biochemical, and biophysical analyses. Our results demonstrate that lutein exerts potent antiviral activity against IAVs by multi-targeting mechanisms including, virucidal activity, inhibition of HA and NA activity, and suppression of viral replication in cells. *In vitro* cytotoxicity assays confirmed that lutein is well-tolerated in MDCK cells at concentrations up to 100 μM for 72 h (Fig. 1). DPPH assays revealed that lutein has minimal radical-scavenging activity compared to standard antioxidants. These findings suggest that lutein’s antiviral effects are not primarily mediated by antioxidant properties, but rather through direct interactions with viral components, particularly the viral envelope and surface glycoproteins.

Virucidal activity assay revealed dose-dependent antiviral effects of lutein. Pre-treatment of A/MA81 with lutein resulted in complete inactivation at 100–200 μM within 30 min, whereas A/PR8 required longer incubation or higher concentrations for complete inactivation (Fig. 2). Lutein also inactivated IBV and achieved complete inactivation of JEV at low micromolar concentrations, while non-enveloped rotavirus showed negligible susceptibility. These observations indicate that lutein preferentially targets lipid-enveloped viruses, likely by affecting viral particle integrity. Mechanistic studies revealed that lutein interferes with viral attachment step, particularly through HA-mediated receptor binding. Co-treatment and virus attachment inhibition assays demonstrated dose-dependent inhibition of viral attachment, and HI assays confirmed that lutein blocks HA-mediated receptor binding at 25–200 μM (Fig. 4). Additionally, virus-coating ELISA showed weak interaction of lutein with A/PR8 HA, further supporting the interaction between lutein and HA. Beyond virus attachment inhibition, lutein also impairs viral release step. Post-treatment experiments showed dose-dependent reductions in CPE and GFP expression for both A/MA81 and A/PR8 viruses (Fig. 5). While lectin-based ELISA revealed that lutein potently inhibits NA enzyme activity in a concentration-dependent manner, the MUNANA assay, which measures direct active-site inhibition, indicated only modest NA inhibition activity of lutein (Fig. 6). This discrepancy suggests that lutein may not directly block the catalytic site of NA, but rather alters glycoprotein accessibility or virion surface architecture, indirectly impairing NA enzymatic activity.

DLS analysis provided complementary evidence that lutein compromises the structural integrity of influenza virions (Fig. 7). Lutein treatment led to dramatic increases in hydrodynamic diameter, elevated polydispersity indices, and heterogeneous multimodal distributions for both A/MA81 and A/PR8 viruses, accompanied by significant decreases in intact particle concentrations. These findings indicate that lutein induces virion aggregation and membrane disruption, consistent with a direct virucidal effect on the viral envelope. Such disruption may also contribute to the inhibition of HA- and NA-mediated functions by altering glycoprotein conformation and accessibility. The preferential activity against enveloped viruses supports the hypothesis that lutein integrates into or interacts with lipid bilayers, altering membrane fluidity and glycoprotein accessibility. Several natural products have been reported to exert antiviral activity through direct interactions with the viral envelope, thereby impairing membrane integrity. For example, labyrinthopeptins such as LabyA1 and LabyA2 target phosphatidylethanolamine within viral membranes and induce virolysis, leading to potent inhibition of enveloped viruses, including Zika virus [34]. Similarly, plant-derived pigments such as pheophorbide a have been shown to rigidify viral membranes in a light-dependent manner, effectively suppressing the entry of coronaviruses [35]. Other natural compounds, including isobavachalcone and corosolic acid, have also been reported to disrupt viral membrane assembly or alter membrane fluidity, thereby reducing infectivity [36]. These findings highlight that lipid-targeting natural products represent an important but relatively underexplored class of antivirals. In this context, our study is the first to demonstrate that lutein exerts antiviral activity against influenza viruses by directly altering viral particle size distribution and abundance, suggesting that modulation of the viral lipid envelope may underlie its inhibitory effects. Although the extent to which lutein’s lipid-targeting properties influence HA and NA functions remains to be elucidated, this observation not only broadens the repertoire of membrane-targeting natural antivirals but also underscores the potential of lutein as a novel scaffold for the development of broad-spectrum antivirals against influenza and other enveloped viruses such as JEV.

Analysis of viral growth kinetics supported lutein’s dual mechanism of action. Pre-treatment to cells of lutein reduced viral titers significantly for both A/MA81 (20.5-fold) and A/PR8 (4.2-fold) at 24 hpi, highlighting its prophylactic potential (Fig. 8). Although our study primarily focused on the direct virucidal and extracellular antiviral effects of lutein, its potential antiviral activity within host cells remains to be elucidated. It is possible that lutein may also modulate intracellular processes such as viral RNA synthesis, protein translation, or host immune signaling pathways, as demonstrated in previous studies [37-39]. Further investigations using intracellular assays and molecular approaches will be essential to determine whether lutein exerts additional antiviral effects. Such studies will provide a more comprehensive understanding of its full antiviral potential and therapeutic relevance.

It is noticeable that lutein consistently exhibited stronger antiviral activity against the A/MA81 compared with A/PR8 strain. This subtype-dependent difference may reflect the combined influence of multiple viral and experimental factors. One plausible explanation is that the lipid composition of the viral envelope may vary between subtypes, thereby altering the interaction with lipophilic compounds such as lutein [40]. In addition, structural differences in HA and NA—including receptor specificity (α2,3-linked sialic acids in H5 versus α2,6-linked sialic acids in H1) and the degree of glycosylation—may affect the accessibility of lutein to the viral surface and its ability to interfere with HA- and NA-mediated functions [41-43]. Finally, H5 viruses often display filamentous particle morphology compared with H1 viruses, which could further influence the impact of lutein on membrane fusion or viral release [44]. Future studies involving lipidomic and glycoproteomic analyses of purified virions, as well as functional assays of HA and NA activity, will be required to clarify the precise mechanisms underlying the higher susceptibility of H5N2 strain to lutein.

Despite the promising *in vitro* efficacy demonstrated in this study, several issues remain to be addressed to establish the *in vivo* relevance of lutein. Considering lutein’s lipophilic nature, future studies should explore optimized delivery systems, such as nanoemulsions or liposomes, to enhance pharmacokinetics and tissue distribution *in vivo*. Structural studies using cryo-electron microscopy or molecular docking could provide direct insights into lutein interactions with viral envelopes or glycoproteins. Critically, *in vivo* evaluation in animal models of influenza infection is required to confirm therapeutic potential, including assessments of viral load reduction, symptom alleviation, and safety profiles. In conclusion, our findings establish lutein as a potent virucidal agent against influenza viruses, functioning through direct virion disruption and inhibition of viral entry, release, and replication. These results highlight lutein’s potential as a novel antiviral candidate and provide a mechanistic framework for future therapeutic development targeting lipid-enveloped viruses.

## Supplemental Materials

Supplementary data for this paper are available on-line only at http://jmb.or.kr.
