# Supplementary material for: Plant-Derived Carotenoid Lutein Demonstrates Multifunctional Antiviral Activity against Influenza A Virus in vitro
Source: J Microbiol Biotechnol. 2025 Nov 18;35:e2507017. doi: 10.4014/jmb.2507.07017 (PMC12640769; doi:10.4014/jmb.2507.07017)
Supplement: Supplementary file 1 [file jmb-35-e2507017-supple.pdf]

J. Microbiol. Biotechnol. 2025. 35: e2507017

https://doi.org/10.4014/jmb.2507.07017

Supplementary Table. S1 Primer sequences for qRT-PCR.

Gene	Direction	Sequences (5' → 3')
GAPDH	Forward	5' - TTC CAC GGC ACA GTC AAG - 3'
	Reverse	5' - ACT CAG CAC CAG CAT CAC - 3'
A/PR8 (H1N1) PB1	Forward	5' - TCA TGA AGG GAT TCA AGC CG - 3'
	Reverse	5' - GGA AGC TCC ATG CTG AAA TTG - 3'
A/PR8 (H1N1) NP	Forward	5' - AGG CAC CAA ACG GTC TTA CG - 3'
	Reverse	5' - TTC CGA CGG ATG CTC TGA TT - 3'
A/PR8 (H1N1) M1	Forward	5' - CGG TCT CAT AGG CAA ATG GT - 3'
	Reverse	5' - CAA CCT CCA TGG CCT CTG CT - 3'
